# Inclusive health for people with disabilities in Chile: a national health system assessment

**DOI:** 10.1186/s12961-024-01241-4

**Published:** 2025-02-20

**Authors:** Danae Rodríguez Gatta, Constanza Piriz Tapia, Elvira Tagle Schmidt, Jimena Luna Benavides, Daniel Vivar Jara, Rodrigo Moreno Celis, Gonzalo Tobar Carrizo, Judit Vilaró Cáceres, Phyllis Heydt, Lena Morgon Banks, Hannah Kuper

**Affiliations:** 1 International Centre for Evidence in Disability, London School of Hygiene and Tropical Medicine, London, WC1E 7HT United Kingdom; 2 Department of Rehabilitation and Disability, Ministry of Health, Santiago, Chile; 3 Center for Development of Inclusive Technologies (CEDETi), Pontifical Catholic University of Chile, Santiago, Chile; 4 Corporación para la Inclusión de Personas con Discapacidad Visual y Sordociegas (CIDEVI), Santiago, Chile; 5 Red Nacional de Fibromialgia, Santiago, Chile; 6 Agrupación Lupus, Santiago, Chile; 7 Disautonomía Chile, Santiago, Chile; 8 Missing Billion Initiative, Seattle, United States of America; 9 Fundación Fibromialgia en Acción, Santiago, Chile; 10 Federación Chilena de Enfermedades Raras (FECHER), Santiago, Chile

**Keywords:** Health policy and systems research, People with disabilities, Disability-inclusive health, Chile

## Abstract

**Background:**

Globally, one in six people have disabilities. They often experience health inequities and many of them arise from system-level failures. This study aimed to assess the inclusion of people with disabilities in the health system of Chile and define recommendations for improvement on the basis of the evidence.

**Methods:**

A health system assessment was conducted between June and November 2023 following the Missing Billion Disability-Inclusive Health Systems Framework and System Level Assessment Toolkit. The assessment was led by the Ministry of Health and conducted by a task team, including organizations of people with disabilities. Mixed methods were used to collect data on nine system-level and service delivery components for a set of 33 indicators, including through a health policy review, systematic review, key informant interviews and a scoping review. Scores were assigned to indicators, components and the overall health system. With this assessment, key recommendations were developed and agreed upon on the basis of a prioritization analysis of impact and feasibility during workshops.

**Results:**

The Chilean health system was assessed to have a low progress towards disability-inclusive health. Among system-level components, intermediate progress has been made in governance, health financing and data and evidence. However, progress in leadership on disability seems low. Among service delivery components, the accessibility of health facilities and rehabilitation and assistive technology showed the best results. However, there were notable gaps in the autonomy and awareness and ability to afford care by people with disabilities, and the capacity of human resources to support this group. The task team defined priority actions in governance, leadership, and human resources.

**Conclusions:**

Short-term actions for the country should involve foundational governance on inclusive health, strengthened leadership of people with disabilities, and mandatory training of healthcare workers to improve healthcare access among this population. Future reassessments should be conducted to monitor and evaluate progress on effective healthcare coverage and health status among people with disabilities.

**Supplementary Information:**

The online version contains supplementary material available at 10.1186/s12961-024-01241-4.

## Background

Globally, one in six people have disabilities [1]. According to the United Nations Convention on the Rights of Persons with Disabilities (UNCPRD), they include “those who have long-term physical, mental, intellectual or sensory impairments which in interaction with various barriers may hinder their full and effective participation in society on an equal basis with others” [2]. Global evidence demonstrates that people with disabilities frequently experience health inequities [1, 3], including a 10–20-year mortality gap [1, 4]. They often experience increased morbidity, with more than double the prevalence of diabetes, cerebrovascular disease, or depression [1]. They also frequently require disability-related services, such as rehabilitation and specialist services [3]. Consequently, people with disabilities can be described as having greater healthcare needs, although they often face systemic barriers to receiving required care.

Health inequities are an important concern in the Americas region, which also has one of the highest prevalences of disability worldwide (19%) [1]. Chile is a high-income country of nearly 20 million people with an increasingly ageing population [5, 6], including approximately 3 million people with disabilities (18%) [7]. A recent literature review of Latin America and the Caribbean (LAC) showed that people with disabilities use health services more frequently than those without disabilities, yet gaps remain in the coverage, affordability, and quality of healthcare due to access barriers [8]. Addressing these gaps is essential for the advancement of the right to health and universal health coverage, as well as making better healthcare for all [1, 3].

**Fig. 1:**
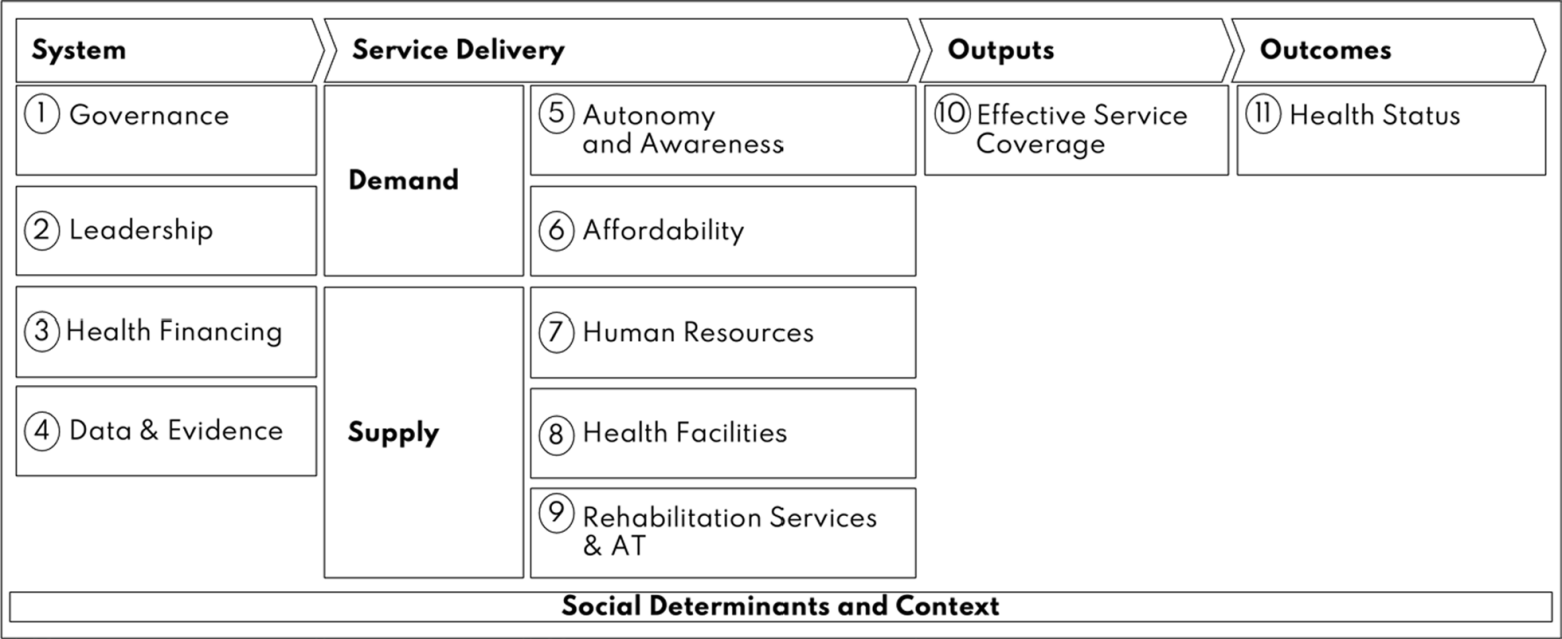
Missing Billion Inclusive Health Systems framework (Source: Missing Billion Initiative and Clinton Health Access Initiative, 2022; Reimagining health systems that expect, accept and connect 1 billion people with disabilities. Available at: https://www.themissingbillion.org/mb-report-2022)

Disability-inclusive health means that people with disabilities have the same access to the full range of health services (e.g. prevention, promotion, treatment) as people without disabilities, in line with the human rights model of disability. Thus, to realize disability inclusion in the health sector, the rights and meaningful participation of people with disabilities should be ensured, as well as health services intentionally designed to “expect, accept, and connect” them to quality care [3, 9]. Health systems therefore need to be strengthened to include people with disabilities, such as through improving health policies, leadership on disability in the Ministry of Health (MoH), financing of inclusive health, or appropriate training of the health workforce [1, 3]. However, current approaches to assess health systems to identify where action is needed have not been designed to focus on disability [10, 11]. Therefore, the Missing Billion Disability-Inclusive Health System Framework and System Level Assessment Toolkit were developed to support MoHs to evaluate the extent of disability inclusion in their health system and identify potential areas for improvement (Fig. 1) [3, 12]. The framework is based on the WHO Building Blocks [10] and Primary Health Care Performance Initiative framework [11], with additional emphasis on components needed to enable disability inclusion [3, 12]. It includes system-level components on governance, leadership on disability within the MoH and representation of people with disabilities, financing of inclusive health, rehabilitation and assistive technology (AT), as well as data and evidence about disability and health. It also has service delivery components across the demand and supply side of healthcare: autonomy and awareness of people with disabilities, affordability of healthcare, health worker training on disability, accessibility of health centres, and availability of rehabilitation services and AT. The framework has an accompanying indicator set to allow for assessment of inclusion for each of the framework components. The framework and indicators were reviewed by a range of experts (governmental and UN stakeholders, health systems specialists, academics, and disability rights organizations) and pilot-tested in the Maldives and Zimbabwe [12].

The aim of this study is to undertake an assessment of the inclusion of people with disabilities in the health system of Chile and define recommendations for improvement on the basis of the evidence.

## Methods

### Study design

A health system assessment was carried out between June and November 2023 following the Missing Billion Disability-Inclusive Health Systems framework (Fig. 1) and System Level Assessment Toolkit [3, 12]. Mixed methods were used to collect data for a set of indicators related to components of the framework, including a health policy review, systematic review, key informant interviews, scoping review of grey and scientific literature, and population-based data. Workshops were held to agree on recommendations and priority actions.

### Study team

The assessment was conducted in Chile and led by the Department of Rehabilitation and Disability of the Ministry of Health of Chile and London School of Hygiene and Tropical Medicine. The MoH convened a task team to conduct the assessment of 11 members, including government representatives (*n* = 2), academia (*n* = 1), and civil society (*n* = 8) (Additional Table 1). All organizations of people with disabilities (OPDs) engaged in a voluntary role and had previously participated in advisory roles at ministerial or parliamentary levels.

**Table 1:** Framework components and number of indicators

Component	Description	Number of indicators
1. Governance	Appropriate in-country laws and policies assert the right to reasonable accommodation and outlaw discrimination on the basis of disability	6
2. Leadership	Disability is clearly articulated and represented in the Ministry of Health, health sector structures and coordination mechanisms	3
3. Health financing	There is sufficient earmarked disability inclusion, assistive technology and rehabilitation budget	3
4. Data and evidence	Data showing the health situation of people with disabilities, evidence to understand and improve health services	4
5. Autonomy and awareness	People with disabilities make their own decisions about healthcare and are aware of their rights and options	3
6. Affordability	People with disabilities can afford to access healthcare	4
7. Human resources	Health workforce is knowledgeable about disabilities and has the skills and flexibility to provide quality care	5
8. Health facilities	Healthcare services, including healthcare facility infrastructure and information, are accessible for people with disabilities	2
9. Rehabilitation services and assistive technology	Rehabilitation and specialist services are available, affordable and of good quality for people with disabilities	3

Source: Missing Billion Initiative and Clinton Health Access Initiative (2022) Reimagining health systems that expect, accept, and connect 1 billion people with disabilities. Available at: https://www.themissingbillion.org/mb-report-2022 (Accessed: 29 June 2023)

### Study setting

Chile has a dual health system mainly based on a public health insurance scheme provided by the National Health Fund (FONASA), covering healthcare for about 79% of the population, and the Private Health Insurances (ISAPRES) covering around 16% [13, 14]. All workers pay compulsory health contributions (7% of their income) into FONASA or ISAPRES [15]. FONASA covers all workers (formal or informal), pensioners and those without income, as well as their legal dependents, regardless of age, gender, income level, health state, or nationality [16]. Health services are delivered by both public and private providers, and the public health network is mostly state funded [13, 16]. About 88% of people with disabilities in Chile are covered by FONASA [7].

### Data collection

We collected data for a set of 33 indicators across 9 framework components: 16 in the system-level and 17 in the service delivery domains (Table 1). Each indicator included a definition, metric and scoring logic (Table 2 and Additional Table 2).

**Table 2:** Health system assessment results per indicator

Component	Indicator	Definition	Indicator score	Component score
Governance	1.1 UNCRPD	Ratification and adoption of UNCRPD	1 − Ratified and evidence of action	0.7
	1.2 National Law	Existence of a national law protecting the right to health for people with disabilities	1 − National law exists that prohibits discrimination and requires reasonable accommodations	
	1.3 National Health Policy or Decree	Existence of a national policy or decree on health for people with disabilities	1 − National decree exists, ensuring access to general healthcare, specialists and measures for implementation	
	1.4 National Health Sector Plan(s)	Inclusion of people with disabilities in National Health Sector Plan(s)	0.2 − National Health Sector Plan includes people with disabilities	
	1.5 National Disease Plan(s)	Inclusion of people with disabilities in National Disease Plan (e.g. HIV, hepatitis)	0 − No	
	1.6 Cross ministry governance	Cross-ministry structure to coordinate work on disability inclusion	1 − Structure exists, including the MoH	
Leadership	2.1 MoH leadership	Existence of a focal point/team in MoH that is responsible for ensuring health access for people with disabilities	1 − Yes	0.3
	2.2 National health sector coordination	National health sector with formal representation of people with disabilities in highest-level	0 − No	
	2.3 Pandemic preparedness structures	Formal representation of people with disabilities in national taskforce	0 − No	
Health financing	3.1 Disability inclusion budget	Budget for department in MoH working on disability inclusion	1 − Yes, at the central level	0.7
	3.2 Reimbursement adjustments	Reimbursement adjustments available for services provided to patients with disabilities	0 − No	
	3.3 Rehabilitation/AT budget	Funding for rehabilitation/AT in MoH budget	1 − Yes	
Data and evidence	4.1 Maturity of disability and health data collection	Health information records tag people with disabilities (electronic integrated system)	0.33 − Data are collected through national surveys	0.7
	4.2 Quality of disability and health data collection method	(a) Data collection method is valid (b) Data collection is recent (in < 10 years) (c) Data are nationally representative (d) 5+ impairment types are covered	1 − Yes	
	4.3 Maturity of disability and health data usage	Data collected are analysed, published and used to direct policy change	0.5 − Data are analysed and published	
	4.4 Quality of disability and health data usage method	(a) Method is transparent and valid (b) Data are analysed and published within 3 years of collection and (c) the analysis is nationally representative (d) Publications and raw data are easily accessible	1 − Yes	
Autonomy and awareness	5.1 OPDs advocacy	OPDs advocate on the right to health for people with disabilities with government	1 − Yes, with the MoH	0.3
	5.2 Autonomy and awareness	People with disabilities report autonomy and awareness about health access	0 − Not reported	
	5.3 Accessibility of health information	Health information is available in accessible formats	0 − No, there are less than two accessibility formats available	
Affordability	6.1 Health coverage	People with disabilities are fully covered for free healthcare	0.5 − Partial coverage	0.3
	6.2 Disability transport subsidy	Transport subsidy is available, including travel to medical care	0 − No	
	6.3 Disability allowance	Available to cover healthcare fees not covered by existing insurance to people with moderate-to-severe disabilities	0.5 − For some people with disabilities	
	6.4 Co-payments	Any co-pays for services in either health insurance or taxation-based systems are waved for people with disabilities	0 − For some people with disabilities and health providers	
Human resources	7.1 Training of medical doctors	Information about disability delivered as part of the national curricula for medical schools/colleges	0 − No	0.1
	7.2 Training of nurses	Information about disability delivered as part of the national curricula for nurses/nursing colleges	0 − No	
	7.3 Training of CHWs	Information about disability delivered as part of the national CHW training curricula	0.33 − Voluntary training with some content covered	
	7.4 Representation in health workforce	People with disabilities are represented in the health workforce	0 − Representation is below 4%	
	7.5 Satisfaction	People with disabilities report that they feel well treated by health workers	0 − Not reported	
Health facilities	8.1 National accessibility standards	Existence of national accessibility standards for healthcare facilities	1 − Yes	0.7
	8.2 Accessibility of facilities	Accessibility audit of health facilities has been undertaken in the last 10 years	0.33 − Local accessibility audit	
Rehabilitation and AT	9.1 National assessments	National assessment of AT/rehabilitation conducted in the last 10 years	0 − No	0.7
	9.2 Cross-ministry coordination for rehabilitation and AT	Coordination mechanism cross-Ministry for rehabilitation services and AT where more than one ministry is involved	1 − Yes	
	9.3 Trained workforce	Physiotherapists available and trained to provide rehabilitation services and AT	1 − Yes	

*AT* assistive technology, *CHWs* community health workers, *MoH* Ministry of Health, *OPDs* organizations of people with disabilities, *UNCRPD* United Nations Convention on the Rights of Persons with Disabilities.

For instance, the first governance indicator consists of the ratification and adoption of the UNCRPD, and its metric requires evidence of it being actioned (e.g. dedicated budget, action plans, and initiatives). The indicators were translated into Spanish and the translation was revised by an external assessor. The following sources of data were collated, across the indicators:

- **Health policy review:** 13 national health policy documents were reviewed. Policies must have been in place at the national level and impact the provision of health services for people with disabilities [17]. Eligible documents were searched through official websites of the MoH [18], Ministry of Social Development and Family [19] and the library of the National Congress of Chile [20].
- **Systematic review:** Peer-reviewed scientific articles of quantitative research about healthcare access among people with disabilities (utilization, coverage, quality, and affordability of healthcare), published since 2000 in Latin America and the Caribbean, were searched in EMBASE, MEDLINE, LILACS, MedCarib, PsycINFO, SciELO, CINAHL, and Web of Science [8].
- **Scoping review:**
- Grey literature, including public or internal government and civil society reports sought through official government websites and the database of the Committee on the Rights of Persons with disabilities [21].
- Peer-reviewed scientific articles published in the last 10 years. Search strategies included keywords of the indicator set and were developed in Spanish and English using relevant databases (SciELO, EMBASE, MEDLINE).
- Publicly available reports of national population-based surveys, conducted in the last 10 years, on disability, healthcare and socio-economic characterization, disaggregated by disability, sought on the website of the Department of Epidemiology of the MoH [22] and in the Social Observatory of the Ministry of Social Development and Family [23].
- **Key informant interviews:** The lead researcher interviewed 20 key national stakeholders, either in person or via Zoom. A purposive sampling was applied to ensure representation of areas of expertise across the framework components. Participants were recruited through recommendations of the task team and snowball sampling was applied throughout the interviews. Informants included government officials (directors, head of departments, policy officers), academic experts with and without disabilities, and OPDs (Table 3). Semi-structured interview guides focussing on each framework component were used. Interviews lasted between 45 min and 60 min and were audio-recorded.

**Table 3:** Participants of key informant interviews (*n* = 20)

Sector	Department, institution
Government (*n* = 11)	(1) Life Cycle Department, MoH
	(2) Rehabilitation and Disability Department, MoH
	(3) Cabinet, Subsecretariat of Public Health, MoH
	(4) Division for Disease Prevention and Control, MoH
	(5) National Commission on Preventive Medicine and Disability, MoH
	(6) Emergency and Disaster Risk Management Department, MoH
	(7) Care Management Department, MoH
	(8) Social Welfare Institute, Ministry of Labour and Social Security
	(9) National Office, National Disability Agency, Ministry of Social Development and Family
	(10) Evaluation and Studies Department, National Disability Agency, Ministry of Social Development and Family
	(11) Health Department, National Board for Student Aid and Scholarships, Ministry of Education
Civil society (*n* = 5)	(12) National Organization of People with Disabilities
	(13) National Organization for Independent Living
	(14) International Organization for the Deaf
	(15) National Organization of People with Autism Spectrum
	(16) National Organization for Women with Disabilities
Academia (*n* = 4)	(17) Sociology School, Diego Portales University
	(18) Public Health School, University of Chile
	(19) Chilean Association of Medical Education
	(20) Chilean Association of Nursing Education

*MoH* Ministry of Health

### Data analysis

#### Scoring of indicators

**Fig. 2:**
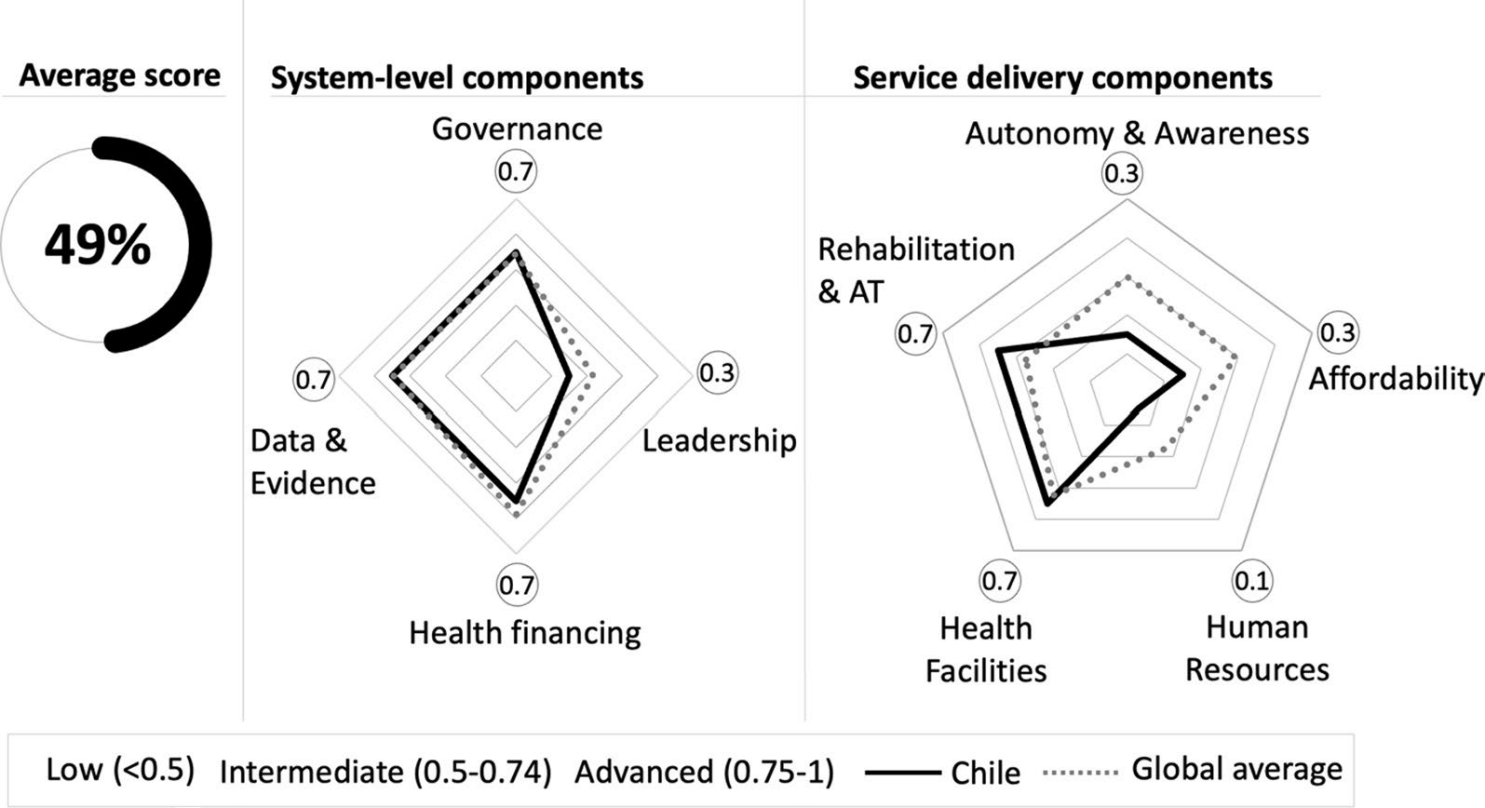
Average scores of the Chilean health system by system-level and service delivery components

Interviews were transcribed and qualitative descriptions were made of the transcripts against the corresponding framework components. Information from the different data sources – peer-reviewed, grey literature, and public records – were then triangulated, validating, enlarging, and articulating information from interviews with documentary sources [24]. The task team held monthly sessions to collectively assess preliminary responses to indicators, identify additional sources of information, and agree on final scoring of indicators. Scores were assigned to each indicator on the basis of the evidence available, ranging from 0 (lowest; no criteria met or evidence of inclusion) to 1 (maximum; all criteria met) [12] (Additional Table 2). Thereafter, each framework component was assigned a score on the basis of the average score of its indicators. The average score was categorized as low (below 0.5), intermediate (between 0.5 and 0.74), or advanced (between 0.75 and 1). Finally, an overall score was calculated for the health system on the basis of the average of its components (each weighted equally). A global average score of other countries (Brazil, Maldives, Zimbabwe, Singapore, Uganda, Australia, United Kingdom, France, and South Africa, amongst others) was available for reference (Fig. 2) [12].

#### Recommendations and priority actions

**Fig. 3:**
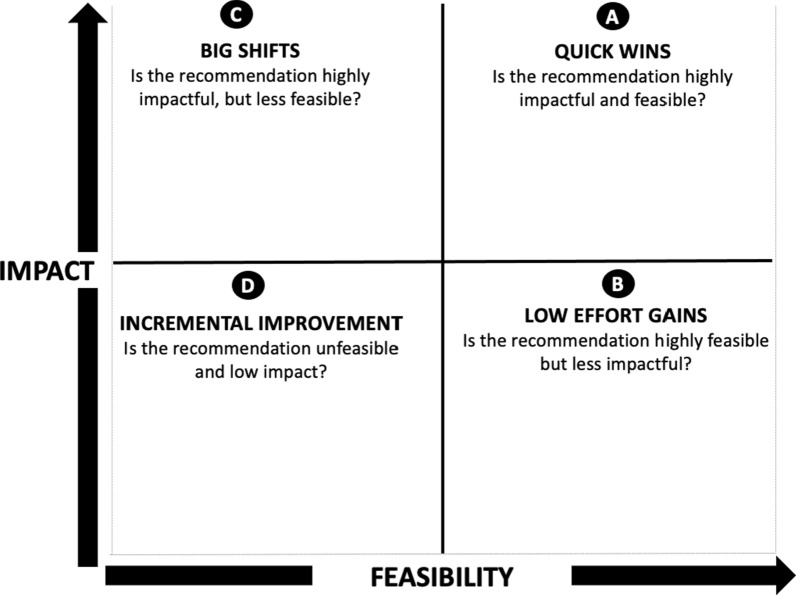
Prioritization matrix based on impact and feasibility criteria (Source: Missing Billion Initiative, 2023; Missing Billion Toolkit – System Level Assessment. Available at: https://www.themissingbillion.org/system-assessment)

The lead author developed provisional recommendations for all indicators that obtained scores below one. Additional emergent recommendations were added from the task team and key informant interviews. Then, all provisional recommendations were assessed on the basis of their potential for impact and feasibility. Criteria of impact included: (1) foundational importance, (2) opportunity for improvement, (3) number of people with disabilities benefited from the intervention and (4) time to impact. Criteria of feasibility included: (1) time to implementation, (2) cost, (3) stakeholder, and (4) technical complexity [12]. The MoH assigned a score to each criterion on the basis of their technical expertise, ranging from one (low) to three (high). Thereafter, an average score of impact and feasibility criteria was calculated for each recommendation. A high average score was two or above, whereas a low score was below two. Finally, all provisional recommendations were distributed in a prioritization matrix by level of impact and feasibility (Fig. 3) [12].

A total of three half-day workshops (one in person and two virtual) were held with the task team to review the assessment’s findings and agree on key priority actions for improvement. The task team discussed the relevance and appropriateness of the provisional recommendations and their distribution in the prioritization matrix in the context of Chile. Subsequently, amendments were made according to the discussions and a final list of recommendations was consolidated. Ultimately, three main priority actions were agreed.

### Ethical approval

This study obtained ethical approval by the Ethics Committee of the authors’ institute.

## Results

The health system in Chile, with respect to disability-inclusive health, obtained an overall low average score of 49% (Fig. 2).

### System-level components

#### Governance

##### UNCRPD (score = 1)

Chile ratified the UNCRPD in 2008 and subsequently adopted specific measures for action [e.g. it created the national disability law no. 20.422, restructured the National Disability Agency (SENADIS) of the Ministry of Social Development and Family, and expanded the Rehabilitation Program] [25, 26].

##### National Law (score = 1)

Law no. 20.422, Establishing Rules on Equal Opportunities and Social Inclusion of Persons with Disabilities, prohibits discrimination in health and demands the implementation of reasonable accommodations for people with disabilities [27]. Additional disability-related laws exist, which protect access to healthcare for people with disabilities. For instance:

- Law no. 20.584, Regulates the rights and duties of individuals in relation to actions related to their healthcare [28].
- Law no. 21.331, On the recognition and protection of the rights of persons in mental healthcare [29].
- Law no. 21.545, Establishes the promotion of inclusion, comprehensive care and the protection of the rights of persons with autism spectrum disorder in the social, health and educational spheres [30].

##### National health policy or decree (score = 1)

Currently, there is no national policy on inclusive health for people with disabilities. However, National Supreme Decree no. 2 approves the regulations governing the right to preferential care [31]. It guarantees priority access for people with disabilities to appointments for primary care, specialists, emergencies, medicines and examinations, and establishes measures for its implementation.

##### National health sector plan(s) (score = 0.2)

The National Health Strategy 2030 includes objectives for functioning and disability [32]. It prioritizes specific health conditions, including childhood developmental disorders, rare diseases, musculoskeletal disorders, autism spectrum disorders, rheumatoid arthritis, and severe dependency. However, it does not include actions and targets for general healthcare and specialist services for all people with disabilities. It also does not include basic statistics about people with disabilities and health.

##### National disease plans (score = 0)

National plans exist for certain diseases [e.g. human immunodeficiency virus/acquired immunodeficiency syndrome (HIV/AIDS), cancer, silicosis, etc.], and although these plans are described as universal, in some cases, certain groups are prioritized. For instance, the National Plan for the Prevention and Control of HIV/AIDS targets only migrants and Indigenous peoples [33]. However, the plan does not explicitly mention people with disabilities to ensure their access to testing, treatment and information programs.

##### Cross ministry governance (score = 1)

Law no. 20.530 established the Interministerial Committee on Social Development and Family. It is chaired by the Ministry of Social Development and Family and includes the participation of the MoH [34]. The committee advises on the government’s social policy and facilitates coordination, guidance, information and agreement among its members, including on disability issues. There is collaboration between the MoH and SENADIS in the certification and qualification of disability, provision of AT and implementation of Law no. 21.545 on people with autism spectrum disorder [26]. However, this collaboration does not occur for inclusive health for all people with disabilities. Furthermore, there is no technical counterpart in SENADIS with an exclusive role in healthcare access.

#### Leadership

##### MoH leadership (score = 1)

Leadership on disability inclusion is diffused and different teams address disability-related issues within the MoH. The Department of Rehabilitation and Disability of the Subsecretariat of Public Health was considered as the lead on disability inclusion by interviewees. The department endorses disability inclusion, although its stated role focusses on disability prevention and habilitation and rehabilitation strategies, not on general healthcare for people with disabilities [35]. This department has historically addressed only the needs of people with physical and sensory disabilities, while the Department of Mental Health has addressed the needs of persons with psychosocial disabilities [36]. Additional teams that address disability-related issues include the National Commission of Preventive Medicine and Disability (COMPIN) and the rehabilitation officers of the Division of Healthcare Network Management and the Division of Primary Care.

##### National health sector coordination (score = 0)

There is no national health sector coordination with formal representation of people with disabilities at the highest level. Current temporary participation occurs for certain health conditions and mental health services, but not on overarching disability-related issues. For example, the ENLACE task team includes representatives of the MoH and organizations of people with autism to implement the new law on autism. As another example, some people with psychosocial disabilities participate in the Mental Health Advisory Council 2022–2024 [37] and in the National Commission for the Protection of the Rights of Persons with Mental Illness [38].

##### Pandemic preparedness structures (score = 0)

The National Pandemic Response Commission COVID-19 is made up of external scientific advisors, technical teams from the MoH, and an inter-ministerial committee [39, 40]. Although civil society could participate, no formal representation of people with disabilities exists. However, SENADIS led a temporary Intersectoral Taskforce on Disability and COVID-19 with representation of people with disabilities [41]. The taskforce developed recommendations for the care of people with disabilities in health services during the coronavirus disease 2019 (COVID-19) pandemic [42].

#### Health financing

##### Disability inclusion budget (score = 1)

The Department of Rehabilitation and Disability of the MoH receives US$ 18 668 per year for governance in rehabilitation, disability prevention, and disability inclusion. However, the budget is considered by interviewees to be insufficient to implement public policies on inclusive health. Furthermore, the Subsecretariat of Healthcare Networks has no budget for the implementation of the law on preferential care for people with disabilities [43].

##### Reimbursement adjustments (score = 0)

There are no health insurance reimbursements or adjusted capitation rates for people with disabilities in FONASA or ISAPRES. However, all beneficiaries of FONASA, including people with disabilities, can apply for reimbursement of expenses associated with the purchase of prostheses and orthoses, or travel associated with the purchase through the public system [44]. It reimburses hip prostheses, cane or tripod, orthopaedic insoles, optical lenses, hearing aids, crutch, rubber heel pad and spinal orthosis.

##### Rehabilitation/AT budget (score = 1)

In 2023, the Subsecretariat of Healthcare Networks of the MoH had an annual budget of about US$ 15 941 million for the Comprehensive Rehabilitation Program in Primary Healthcare. It also had a 2022 annual budget of US$ 38 976 million for the financing of AT through the Explicit Health Guarantees (GES) scheme and the Ricarte Soto scheme, which establishes a system of financial protection for high-cost diagnosis and treatment regardless of health insurance type [45, 46]. In addition, SENADIS had an annual 2023 budget for its AT Program of US$ 6540 million.

#### Data and evidence

##### Maturity of disability and health data collection method (score = 0.33)

The main data collection on disability and health is through population-based surveys [7], including the national disability survey from 2022. Census 2024 will incorporate questions on disability [47]. There is a National Register of Disability, in which in June 2023 only 23% of the population with disabilities (*n* = 625 848) were included [48]. Currently, the register facilitates access to social benefits, but it does not keep integrated statistics with health information of people with disabilities. Furthermore, health information records collect data on disability status in public and private health facilities [49]. These data are mandatory and require the Community Assessment of Performance Evaluation (IVADEC-CIF) by health professionals to determine the origin and extent of disability of the person. However, data collected from health facilities do not include health indicators of people with disabilities [50].

##### Quality of disability and health data collection method (score = 1)

The disability national survey from 2022 is based on the Model Disability Survey, a tool recommended and validated by the WHO, and is nationally representative and disaggregates results by six types of disabilities [7].

##### Maturity of disability and health data usage (score = 0.5)

Data on disability and health collected through national surveys are analysed and published [7]. The data are used to define targets in the national health strategy and for budget allocation. However, only findings related to rehabilitation and AT have been used to guide policy changes, in contrast to general healthcare of people with disabilities [46, 51]. Available statistics on disability and health are currently not harmonized. Consequently, there is a lack of robust figures on the total population with disabilities and their needs at regional/community level.

##### Quality of disability and health data usage method (score = 1)

Data collected on disability and health are analysed and published in public repositories within 1–2 years of collection [7, 52]. The reports describe the methods of data analysis, maintain analyses at national and regional levels and full databases are shared for different statistical software.

### Service delivery components

#### Autonomy and awareness

##### Organizations of people with disabilities advocacy (score = 1)

Some people with disabilities and OPDs have advised the MoH, for instance, through the current ENLACE task team for the implementation of Law no. 21.545 for people with autism or the Mental Health Advisory Council [30, 37].

##### Autonomy and awareness (score = 0)

There are a lack of data on autonomy and awareness of healthcare access for people with disabilities from within the last 10 years from population-based surveys and qualitative data.

##### Accessibility of health information (score = 0)

The Ministry of Health’s website and its partner websites, which are the main sources of online health information, have few accessible formats available [18, 53]. For example, they feature accessibility tools (e.g. text-to-speech function), and some videos include sign language interpretation. However, no accessible formats such as easy-to-read, sign language interpretation on all videos, Braille or information for caregivers are observed, nor do links exist to request the delivery of health information in alternative formats.

#### Affordability

##### Health coverage (score = 0.5)

*Coverage associated with disability:* There are stipulations that guarantee financial coverage for people with certified disabilities. For instance, free healthcare is provided in the public network to people with severe or profound disabilities, under 18 years of age, affiliated to FONASA and belonging to the 60% lowest socio-economic levels through the disability subsidy [54, 55]. There is also an adjustment of coverage for people with disabilities affiliated to FONASA for rehabilitation services (physio, occupational and speech therapy) received outside the public network [56]. This benefit does not modify service fees but eliminates the annual care cap and also applies to ISAPRES beneficiaries.

*Coverage associated with medical diagnoses:* The GES scheme guarantees financial protection for 87 health conditions, some that could lead to disability, including depression, schizophrenia, bipolar disorder, arthritis, Parkinson’s disease, epilepsy, multiple sclerosis, bilateral hypoacusis, refractive errors, systemic lupus erythematosus, and retinopathies [57]. GES also covers orthoses and AT, cataract surgery and COVID-19 rehabilitation. Similarly, the Ricarte Soto scheme covers the diagnosis (in some cases) and treatment of 27 health conditions, some of them possibly associated with disability, such as multiple sclerosis, rheumatoid arthritis, bilateral sensorineural hearing loss, and systemic lupus erythematosus, amongst others [45]. Finally, FONASA launched a diagnosis associated payment voucher for the diagnosis and treatment of people with autism up to 18 years of age outside the public network with fixed service fees [16].

*Universal coverage:* The entire population affiliated to FONASA receives free medical care in the public network [58]. As a result, people with disabilities would have access to free healthcare because they are covered by FONASA and not because they have a disability. However, 12% of people with disabilities are not affiliated to FONASA and thus will not have free access to medical care through this route [7]. Moreover, health coverage is not free if people with certified disabilities choose to receive healthcare outside the public network, either because of access, timeliness, or quality of care. Furthermore, only certain pharmacological treatments are covered by FONASA.

##### Transport subsidy (score = 0)

There is currently no national transport subsidy for people with disabilities in Chile [59]. Some local subsidies exist at regional or municipal level, where vehicles are available for the transport of patients with disabilities, although they typically focus on people with physical impairments.

##### Disability allowance (score = 0.5)

There is a disability subsidy for people under 18 with severe or profound disabilities, of any impairment type, who are among the 60% lowest socio-economic levels of the population [54]. This group receives a monthly monetary benefit of US$ 112 (as of November 2023). This subsidy includes free medical coverage in the public network for FONASA affiliates. Adults with certified disabilities could receive a disability pension (US$ 225) if they belong to the 80% lowest socio-economic groups [60]. However, there is no disability allowance for all people with disabilities in Chile.

##### Co-payments (score = 0)

FONASA beneficiaries, including people with disabilities, have zero co-payments when receiving medical care in the public network [58]. However, this benefit does not apply to care received by private healthcare providers. In addition, 12% of people with disabilities who do not belong to FONASA are exempted from receiving this benefit.

#### Human resources

##### Training of medical doctors (score = 0)

There is no mandatory national training standard on disability for medical schools, including medical and non-medical aspects. Each medical school determines the curriculum for its students, although the Single National Medical Knowledge Test (EUNACOM) would influence the standard of undergraduate training [61]. At present, EUNACOM does not include an exclusive component on disability as such, only health conditions that could result in disability (e.g. mental health disorders, hearing loss, low vision, etc.).

##### Training of nurses (score = 0)

There is no national curriculum for nursing schools; each school determines their own curriculum. However, there is a voluntary National Nursing Examination (ENENF) that could influence the standard of training [62]. The ENENF includes questions on health conditions (e.g. children and adolescents with special healthcare needs) but there is no exclusive content on disability.

##### Training of community health workers (CHWs) (score = 0.33)

The training manual for CHWs of the Primary Healthcare Division of the MoH includes some elements regarding legal regulations and rights of people with disabilities, use of language around disability and OPDs [63]. However, this training is not mandatory.

##### Representation of people with disabilities in health workforce (score = 0)

There are no official records of the number of health workers with disabilities. However, it is estimated that between 0.05% and 3.5% of health workers in hospitals (Coyhaique Regional Hospital, La Florida Dra. Eloísa Díaz Hospital and Peñaflor Hospital) have disabilities, which is lower than expected for the working age population with disabilities (at least 4% for high-income countries, such as Chile) [64].

##### Satisfaction (score = 0)

There are no surveys on user satisfaction or quality of treatment in health facilities that disaggregate data by disability and allow for comparison with the population without disabilities, or qualitative studies in this area.

#### Health facilities

##### National accessibility standards (score = 1)

There are national accessibility standards for the infrastructure of all public spaces, including both public and private health facilities [65–67]. For example, health facilities must have toilets for people with disabilities, ramps, handrails, etc. There are also universal accessibility standards for web systems and websites of state administration bodies [68]. However, there are no mandatory technical national standards for health communication and information, except for the mandatory provision of sign language interpretation and closed captioning [27].

##### Accessibility audit (score = 0.33)

In the last 10 years, the MoH has neither conducted nor commissioned nationally representative accessibility audits of healthcare facilities. However, an independent evaluation in the northern Atacama region of 18 primary healthcare facilities found low levels of accessibility to information and participation [69].

#### Rehabilitation services and assistive technology

##### National assessments of rehabilitation or AT (score = 0)

There is no national assessment of rehabilitation or AT. However, an inter-ministerial taskforce was recently established to design the National System of Assistive Technology with a unified catalogue and register of AT [70].

##### Cross-ministry coordination for rehabilitation services and AT (score = 1)

Currently, there is an inter-ministerial taskforce for the development of a national system of AT in which several ministries participate, including the MoH [70].

##### Trained workforce available to provide rehabilitation services and AT (score = 1)

There are about 19.8 physiotherapists per 10 000 inhabitants in Chile, meeting the standard expected for high-income countries [71]. In addition, there are 6.7 occupational therapists, 9.9 speech therapists, and 40.3 psychologists per 10 000 inhabitants.

### Recommendation and priority actions

A total of 14 recommendations were considered (Table 4) and three priorities were defined and agreed on to progress disability-inclusive health in Chile in terms of governance, leadership, and human resources:

1. Formulate a National Policy on Inclusive Health for People with Disabilities. It was considered important that this policy is both comprehensive and specific to the diverse health needs, has a budget for implementation, adopts an inclusive approach in all health programs, and is led by staff with disabilities and/or with the permanent and binding participation of OPDs in the design, monitoring, and evaluation of its implementation.
2. Ensure formal representation of people with disabilities, including through their OPDs, in the highest-level health sector coordination structure and in pandemic preparedness structures, avoiding silos and duplication of existing participatory bodies; for example, through a permanent advisory committee on disability and all health matters.
3. Establish a mandatory training program on disability, with a human rights perspective and including both medical and non-medical aspects, for health workers (professional, technical and administrative staff) in both public and private health facilities.

**Table 4:** Additional list of recommendations to improve disability-inclusive healthcare in Chile

Component	Description
QUICK WINS	
(1) Health facilities	Establish a mandatory healthcare protocol for people with disabilities, for the public and private sector, with minimum standards of care that: a. alerts the visit of a patient with disabilities and the rights and benefits associated with disability certification b. schedules healthcare with flexible agendas according to the needs of each person and the prevalence of disability in the territory c. requests informed consent and support for decision-making, especially for persons with psychosocial and intellectual disabilities d. ensures accessibility of processes, information and communication (e.g. sign language, plain language, alternative communication, or visual aids)
LOW EFFORT GAINS	
(2) Human resources	Promote cross-sectoral coordination with academia for disability training of undergraduate medical and nursing students, and advocate for the inclusion of disability questions in national exams (EUNACOM and ENENF)
(3) Human resources	Increase the recruitment of people with disabilities in health facilities in collaboration with OPDs, to promote inclusion in the workplace, raise awareness among health facility staff and patients, and reduce discrimination and stigma towards people with disabilities
(4) Health facilities	Encourage the improvement of accessibility and universal design of health facilities (not only infrastructure standards) and the implementation of reasonable accommodations
MAJOR CHANGES	
(5) Data and evidence	Collect data on disability and health from health records, including issues of autonomy and awareness and satisfaction, and link the National Disability Register with health data. Use findings from the data collected to drive program and policy changes
(6) Autonomy and awareness	Ensure that health information issued from all digital information systems and websites of the MoH (subsecretariats, departments, etc.) and its agencies (SEREMIAS, health services, etc.) is available in accessible formats (e.g. easy-to-read, sign language, Braille, etc.) and/or indicate a link to request alternative formats. In addition, create a section on inclusive on the website of the Department of Rehabilitation and Disability of the MoH
(7) Rehabilitation and AT	Review and expand coverage of both physical and mental rehabilitation services for all persons with disabilities in primary healthcare
GRADUAL IMPROVEMENT	
(8) Governance	Include disability-inclusive health goals and actions in the forthcoming National Health Strategy 2030–2040, incorporating disability and health data as well as monitoring and evaluation indicators
(9) Governance	Prioritize people with disabilities in National Disease Plans (e.g. HIV, TB, etc.)
(10) Rehabilitation and AT	Conduct a national evaluation, including cost-effectiveness and impact indicators, of AT and rehabilitation every 10 years, ensuring that it is nationally representative and that findings are published
(11) Health facilities	Conduct a health facility information and communication accessibility audit

This list excludes the three prioritized recommendations which belonged to “quick wins”

*AT* assistive technology, *MoH* Ministry of Health, *ENEF* National Nursing Examination, *OPDs* organizations of people with disabilities, *SEREMIAS* Regional Health Ministry Secretariats, *EUNACOM* Single National Medical Knowledge Test

Additional, but not prioritized recommendations, would be incorporated into the prioritized actions (Table 4). For example, the national policy on inclusive health should include the development of a healthcare protocol for people with disabilities, inclusion of disability targets in the National Health Strategy 2040, and of people with disabilities in national disease plans. Likewise, the training program should include the development and communication of health information in accessible formats (e.g. in web pages, prescriptions, leaflets, educational materials, etc.).

## Discussion

The Chilean healthcare system appears to have made gradual progress towards inclusive health for people with disabilities, but significant gaps remain. Among system-level components, intermediate progress has been made in governance, health financing, and data and evidence. However, progress in leadership on disability in the MoH seems low. Among service delivery components, the physical accessibility of health facilities and rehabilitation services and assistive technology showed the best results. However, autonomy and awareness, affordability, and human resources achieved the lowest scores.

Chile’s intermediate progress on governance, health financing, data and evidence, health facilities, and rehabilitation services and AT is consistent with the results of international outside-in assessments using the Missing Billion framework [12]. Similarly, Chile’s low progress on leadership and human resources is consistent with the global average on these areas. However, in contrast to the general intermediate progress on affordability and autonomy and awareness, Chile has a limited development. However, to date no previous disability-inclusion health systems assessments have been reported in Chile, and globally, other assessments have focussed on mental healthcare. In 2014, the mental health system in Chile was assessed using the WHO Assessment Instrument for Mental Health Systems [72]. The assessment revealed progress in governance, mental health budget, data collection systems, and increased availability of specialized mental health services. However, weaknesses remained in the availability of specialized staff and services for children and adolescents, quality of care, equity (by location, minority groups, and health insurance type) and leadership of users and their families. These findings are consistent with the gaps and strengths found in disability inclusion.

The Missing Billion Framework is an innovative tool that captures essential issues of disability-inclusive health systems and facilitates its replication in other settings. However, given that the framework offers a standard overview of health systems, some nuance is missed. For instance, the focus in Chile remains mainly on rehabilitation and AT for people with disabilities and initiatives on disability-inclusive health are taking place in silos (i.e. across ministries and between ministries and OPDs) [26, 73, 74]. In addition, the actual prioritization of disability inclusion within the MoH appears to be low [17]. Furthermore, the simple fulfilment of the criteria that was applied might not capture the complexity of health systems. For example, despite Chile scoring the highest for the ratification and adoption of the UNCRPD, gaps might remain in its implementation. Shadow reports of civil society have highlighted the lack of implementation on health and rehabilitation rights (e.g. health worker protocols, accessible health information, Chilean Sign Language interpretation services, mental health budget, low coverage of rehabilitation services and AT) [75]. Similarly, some existing legal frameworks expected to protect the right to healthcare are not exempted from criticism. For instance, civil society has also raised competing issues with Law no. 20.584 and Decree no. 570 regarding psychiatric hospitalization and involuntary sterilization, pertaining particularly people with psychosocial and intellectual disabilities [75, 76].

Some limitations exist regarding this assessment. The framework could be further improved, with the revision of a few scoring criteria. Some indicators achieved the highest score, although further improvement could be made in the areas assessed. For example, while the MoH allocates a disability-inclusive health budget, it is largely underfunded, and the scoring criteria of this indicator does not assess budget sufficiency. Furthermore, scoring of the accessibility audit does not mention the scope of the evaluation and the maximum score can still be obtained even if poor accessibility were to be found in health facilities. Similarly, the rehabilitation and assistive technology assessment indicator does not include scoring criteria regarding the availability of AT and the mechanisms for their acquisition. In addition, indicators could specify whether it relates to all people with disabilities or a subset, as eligibility for benefits varies depending on disability type, severity and certification status. Moreover, health financing and affordability indicators should account more for countries with dual health systems and mixed service provision such as Chile. People with disabilities who are not covered by public health insurance can be excluded from financial adjustments despite the additional living costs associated with disability. [1, 77].

Assessments could take greater account on differences amongst people with disabilities (e.g. rural/urban, type of impairment) and direct representation of all disability groups should be strengthened [78]. Confusion on organization types, lack of funding for advisory roles and poor cohesion of the disability movement have been pointed out as barriers in the participation of OPDs in policy processes and should be addressed in the future [78]. Ultimately, guidance could be provided on how to identify and select OPDs to facilitate wider engagement as well as accessible materials and work dynamics (e.g. right disability language, reasonable accommodations, etc.).

This assessment has important strengths. It is the first comprehensive assessment on disability-inclusive health in Chile with participation of civil society. Findings will serve as a disability-inclusive health benchmark both for Chile and globally. It is the first assessment using the Missing Billion Framework in its complete format with MoH and OPD engagement. The collaboration provided exchange and learning experiences on health and disability for all actors, especially OPDs, who gained skills to monitor and evaluate progress in the future. In addition, the task team compounded technical expertise and lived experience of disability. Information was independently assessed by representatives and their organizations, and multiple key national stakeholders were consulted. Finally, the three priorities for action recommended for Chile at this stage are aligned with the WHO measures for the inclusion of disability in health systems [1] and the ownership of the MoH in this assessment could positively impact policy implementation [79].

## Conclusions

Our findings suggest that only some progress has been made towards disability-inclusive healthcare in Chile. Short-term actions for the country should involve foundational governance on this topic, strengthened leadership of people with disabilities and mandatory training of healthcare workers to improve healthcare access among this group. Future reassessments should be conducted to monitor and evaluate progress on effective healthcare coverage and health status among people with disabilities.

## Supplementary Information

Supplementary Material 1.

## Data Availability

The dataset generated during the current study is not publicly available due to the privacy of individuals that participated in the study but is available from the corresponding author on reasonable request.

## Abbreviations

UNCPRD: United Nations Convention on the Rights of Persons with Disabilities
LAC: Latin America and the Caribbean
MoH: Ministry of Health
WHO: World Health Organization
AT: Assistive technology
OPDs: Organizations of People with Disabilities
FONASA: National Health Fund
ISAPRES: Private Health Insurances
SENADIS: National Disability Agency
COMPIN: National Commission of Preventive Medicine and Disability
GES: Explicit health guarantees
IVADEC-CIF: Community Assessment of Performance Evaluation
EUNACOM: Single National Medical Knowledge Test
ENENF: National Nursing Examination

## References

[1] World Health Organization. Global report on health equity for persons with disabilities. Geneva: World Health Organization; 2022.

[2] United Nations. Convention on the Rights of Persons with Disabilities—Articles. 2006 10.1515/9783110208856.203 18348362

[3] Missing Billion Initiative, Clinton Health Access Initiative. Reimagining health systems that expect, accept and connect 1 billion people with disabilities. 2022.

[4] Rotenberg S, Smythe T, Kuper H. Left Behind: modelling the life expectancy disparities amongst people with disabilities in Low and Middle-Income Countries. medRxiv 2023. 2023.07.12.23292565

[5] World Bank. World Bank Country and Lending Groups. 2023. https://datahelpdesk.worldbank.org/knowledgebase/articles/906519-world-bank-country-and-lending-groups. Accessed 19 Jul 2023

[6] Instituto Nacional de Estadísticas. Proyecciones de población. 2024. https://www.ine.gob.cl/estadisticas/sociales/demografia-y-vitales/proyecciones-de-poblacion. Accessed 9 Jan 2024

[7] Rozas Assael F, González Olave F, Cerón Cañoles G, Guerrero Hurtado M, Vergara Henríquez R, Pinto Mora S (2023) III Estudio Nacional de la Discapacidad. Santiago

[8] Rodríguez Gatta D, Rotenberg S, Allel K, Reichenberger V, Banks M, Kuper H. [Pre Print] Access to general health care among people with disabilities in Latin America and the Caribbean: a systematic review of quantitative research. SSRN. 2023. 10.2139/ssrn.4579884. 10.1016/j.lana.2024.100701 PMC10943476 38495313

[9] World Health Organization. Health equity for persons with disabilities: a guide for action. 2024.

[10] World Health Organization. Everybody’s Business: strengthening health systems to improve health outcomes: WHO’s framework for action. Geneva: World Health Organization; 2007.

[11] Veillard J, Cowling K, Bitton A, et al. Better measurement for performance improvement in low- and middle-income countries: the primary health care performance initiative (PHCPI) experience of conceptual framework development and indicator selection. Milbank Q. 2017;95:836–83. 29226448 10.1111/1468-0009.12301 PMC5723717

[12] Missing Billion Initiative. Missing Billion Toolkit - System Level Assessment. 2023. https://www.themissingbillion.org/system-assessment. Accessed 10 Jan 2024

[13] OECD. OECD Reviews of Public Health: Chile. 2019. 10.1787/9789264309593-en

[14] Sánchez M. Análisis Estadístico del Sistema Isapre con Perspectiva de Género. Santiago. 2022.

[15] Missoni E, Solimano G. Towards universal health coverage: the Chilean experience. 2010

[16] FONASA. Cuenta Pública Participativa 2022. Santiago. 2022

[17] Rodríguez Gatta D, Gutiérrez Monclus P, Wilbur J, Hanefeld J, Banks LM, Kuper H. Inclusion of people with disabilities in Chilean health policy: a policy analysis. Int J Equity Health. 2024;23:174. 39198851 10.1186/s12939-024-02259-4 PMC11360718

[18] Ministerio de Salud. Ministerio de Salud. 2024. https://www.minsal.cl. Accessed 18 Jan 2024

[19] Ministerio de Desarrollo Social y Familia. Ministerio de Desarrollo Social y Familia. 2024. https://www.desarrollosocialyfamilia.gob.cl. Accessed 19 Jan 2024

[20] Biblioteca del Congreso Nacional de Chile. Biblioteca del Congreso Nacional de Chile. 2024. https://www.bcn.cl/portal/. Accessed 18 Jan 2024

[21] United Nations Human Rights. UN Treaty Body Database. 2023. https://tbinternet.ohchr.org/_layouts/15/TreatyBodyExternal/Countries.aspx. Accessed 17 Jan 2024

[22] Ministerio de Salud. Departamento de Epidemiología. 2023. http://epi.minsal.cl/. Accessed 18 Jan 2024

[23] Observatorio Social M de DS y F. Encuesta de Discapacidad y Dependencia 2022. 2022. https://observatorio.ministeriodesarrollosocial.gob.cl/endide-2022. Accessed 19 Jan 2024

[24] Green J. Qualitative methods for health research, 4th edition. SAGE, Los Angeles, 2018

[25] Ministerio de Relaciones Exteriores. Decreto 201 promulga la Convención de las Naciones Unidas sobre los Derechos de las personas con Discapacidad y su protocolo facultativo. Poder Ejecutivo, Santiago, 2008

[26] Estado de Chile. CRPD/C/CHL/2-4. Santiago, 2022

[27] Ministerio de Planificación. Ley N^o^20.422 Establece normas sobre Igualdad de Oportunidades e Inclusión Social de Personas con Discapacidad. Cámara de Diputados, Santiago, 2010

[28] Ministerio de Salud. Ley 20.584 regula los derechos y deberes que tienen las personas en relación con acciones vinculadas a su atención en salud. Cámara de Diputados, Santiago, 2012

[29] Ministerio de Salud. Ley N^o^21.331 del reconocimiento y protección de los derechos de las personas en la atención de salud mental. Cámara de Diputados, Santiago, 2021

[30] Ministerio de Salud. Ley N^o^21.545 establece la promoción de la inclusión, la atención integral, y la protección de los derechos de las personas con trastorno del espectro autista en el ámbito social, de salud y educación. Cámara de Diputados, Santiago, 2023

[31] Ministerio de Salud. Decreto 2 aprueba reglamento que regula el derecho a la atención preferente dispuesto en la ley N^o^20.584 . Cámara de Diputados, Santiago, 2020

[32] Departamento Estrategia Nacional de Salud. Estrategia Nacional de Salud para los Objetivos Sanitarios al 2030 . Santiago, 2022

[33] Departamento del Programa Nacional de Prevención y Control del VIH/SIDA y las ITS. Plan Nacional para la prevención y control del VIH/SIDA y las ITS, 2021–2022. Santiago, 2021

[34] Ministerio de Planificación. Ley N^o^20530 crea el Ministerio de Desarrollo Social y Familia y modifica cuerpos legales que indica. Senado, Santiago, 2011

[35] Ministerio de Salud. Departamento de Rehabilitación y Discapacidad. 2024. https://rehabilitacion.minsal.cl/. Accessed 18 Jan 2024

[36] Ministerio de Salud. Misión y visión del Departamento de Salud Mental. 2024. https://diprece.minsal.cl/conozcanos/departamentos/departamento-de-salud-mental/. Accessed 18 Jan 2024

[37] Ministerio de Salud. Resolución Exenta 443 crea comisión especial electora de integrantes del consejo asesor en salud mental . Santiago, 2021

[38] Ministerio de Salud. Decreto 23 crea Comisión Nacional de Protección de los Derechos de las Personas con Enfermedad Mental . Santiago, 2012

[39] Ministerio de Salud. Resolución 473 exenta crea comisión nacional de respuesta pandémica covid-19 y deja sin efecto consejo asesor covid-19 . Santiago, 2022

[40] Departamento de Comunicaciones y Relaciones Públicas. Covid-19 en Chile. Pandemia 2020–2022. Ministerio de Salud, Santiago, 2022

[41] SENADIS. Directora Nacional de SENADIS encabezó sesión de la Mesa Intersectorial Discapacidad y COVID-19. 2020. https://www.senadis.gob.cl/sala_prensa/d/noticias/8326. Accessed 18 Jan 2024

[42] SENADIS, Pontificia Universidad Católica de Chile, Enviada Especial del Secretario General de Naciones Unidas sobre Discapacidad y Accesibilidad, Universidad de Los Andes, Mesa Intersectorial por la Discapacidad Intelectual, Oficina Regional para América del Sur del Alto Comisionado de las Naciones Unidas para los Derechos Humanos. Recomendaciones para la atención a personas con discapacidad en el contexto de la pandemia de COVID-19. 2020. https://www.senadis.gob.cl/sala_prensa/d/noticias/8226/recomendaciones-para-la-atencion-de-personas-con-discapacidad-en-el-contexto-de-la-pandemia-por-covid-19. Accessed 18 Jan 2024

[43] Ministerio de Salud. Programa de Atención Preferencial. 2021. https://www.dipres.gob.cl/597/w3-article-212525.html. Accessed 18 Jan 2024

[44] FONASA. Reembolso de prótesis y órtesis. 2023. https://www.fonasa.cl/sites/fonasa/adjuntos/PROTESIS. Accessed 18 Jan 2024

[45] Ministerio de Salud. Ley 20.850 crea un sistema de protección financiera para diagnósticos y tratamientos de alto costo y rinde homenaje póstumo a Don Luis Ricarte Soto Gallegos. Cámara de Diputados, Santiago, 2015

[46] SENADIS. Informe Final Programa Ayudas Técnicas 2022–2023. Santiago, 2023

[47] Instituto Nacional de Estadísticas. CENSO 2024. 2024. https://www.ine.gob.cl/censo. Accessed 18 Jan 2024

[48] SENADIS. Calificación y certificación de la discapacidad . Santiago, 2021

[49] Departamento de Estadísticas e Información de Salud. Norma Técnica 820 sobre los Estándares de Información de Salud. Santiago, 2023.

[50] Departamento de Estadísticas e Información de Salud. Manual Series de Resúmenes Estadísticos Mensuales. Santiago, 2023.

[51] Departamento Estrategia Nacional de Salud. Resumen Análisis Crítico Temas Estrategia Nacional de Salud 2011–2020. Santiago, 2020

[52] SENADIS. II Estudio Nacional de la Discapacidad. Santiago, 2016.

[53] Ministerio de Salud. Salud Responde. 2023. https://saludresponde.minsal.cl/. Accessed 18 Jan 2024

[54] Chile Atiende. Subsidio de Discapacidad para menores de 18 años. 2023. https://www.chileatiende.gob.cl/fichas/43187-subsidio-de-discapacidad-para-menores-de-18-anos. Accessed 18 Jan 2024

[55] Ministerio del Trabajo y Previsión Social. Ley 20.255 establece reforma previsional. Cámara de Diputados, Santiago, 2008

[56] SENADIS. Beneficios en Prestaciones de Kinesiología, Fonoaudiología y Terapia Ocupacional. 2023. https://www.senadis.gob.cl/pag/568/1857/beneficios_en_prestaciones_de_kinesiologia_fonoaudiologia_y_terapia_ocupacional. Accessed 18 Jan 2024

[57] Salud Responde. AUGE/GES, patologías y garantías. 2022. https://saludresponde.minsal.cl/auge-ges/. Accessed 19 Jan 2024

[58] Gobierno de Chile. Copago 0 – Red Pública de Salud Gratuita. 2022. https://www.gob.cl/copagocero/. Accessed 18 Jan 2024

[59] Ministerio de Transportes y Telecomunicaciones. Ley 20.378 crea un subsidio nacional para el transporte público remunerado de pasajeros. Cámara de Diputados, Santiago. 2009

[60] Chile Atiende. Pensión Básica Solidaria de Invalidez. 2023. https://www.chileatiende.gob.cl/fichas/5178-pension-basica-solidaria-de-invalidez-pbsi. Accessed 6 Feb 2024

[61] EUNACOM. Examen Único Nacional de Conocimientos de Medicina. 2024. https://www.eunacom.cl/. Accessed 18 Jan 2024

[62] ACHIEEN. Examen Nacional de Enfermería. 2024

[63] Fundación EPES, División de Atención Primaria. Manual de Apoyo a la Incorporación de Agentes Comunitarios en Salud. Santiago. 2016

[64] Departamento de Gestión del Riesgo en Emergencias y Desastres. Implementación de la Metodología Inclusión para la Gestión del Riesgo de Desastres en Hospitales. Santiago. 2019.

[65] Ministerio de Vivienda y Urbanismo. Decreto 50 modifica decreto supremo N^o^47, de vivienda y urbanismo, de 1992, ordenanza general de urbanismo y construcciones en el sentido de actualizar sus normas a las disposiciones de la ley n^o^20.422, sobre igualdad de oportunidades e inclusión social de personas con discapacidad. Santiago. 2015

[66] Ministerio de Vivienda y Urbanismo. Decreto 30 modifica decreto supremo N^o^47, de vivienda y urbanismo, de 1992, ordenanza general de urbanismo y construcciones, en el sentido de actualizar diversas disposiciones relacionadas con la normativa de accesibilidad universal en espacios públicos . Santiago. 2021

[67] SENADIS. Informe técnico de accesibilidad . Santiago. 2017

[68] Ministerio Secretaría General de la Presidencia. Decreto 1 aprueba norma técnica sobre sistemas y sitios web de los órganos de la administración del estado. Santiago. 2015.

[69] Campillay-Campillay M, Calle-Carrasco A, Dubo P, Moraga-Rodríguez J, Coss-Mandiola J, Vanegas-López J, Rojas A, Carrasco R. Accessibility in people with disabilities in primary healthcare centers: a dimension of the quality of care. Int J Environ Res Public Health. 2022;19:12439. 36231740 10.3390/ijerph191912439 PMC9564706

[70] SENADIS. Mesa Interministerial continúa trabajando para avanzar a un Sistema Nacional de Ayudas técnicas . 2023. https://www.senadis.gob.cl/sala_prensa/d/noticias/9014/mesa-interministerial-continua-trabajando-para-avanzar-a-una-sistema-nacional-de-ayudas-tecnicas. Accessed 18 Jan 2024

[71] Subsecretaria de Redes Asistenciales. Dotación de personal del sistema nacional de servicios de salud 2023 . Santiago. 2023.

[72] Minoletti A, Alvarado R, Rayo X, Minoletti M. Sistema de Salud Mental de Chile. Santiago. 2014

[73] SENADIS. Balances Históricos de Gestión Integral. Santiago. 2022

[74] SENADIS. Campaña Conversemos Discapacidad y Sexualidad. 2023. https://www.senadis.gob.cl/participacion/d/noticias/9022/campana-conversemos-discapacidad-y-sexualidad. Accessed 18 Jan 2024

[75] United Nations (2020) CRPD/C/CHL/QPR/2–4. List of issues prior to submission of the combined second to fourth reports of Chile. Geneva

[76] Ministerio de Salud. Construyendo Salud Mental. Santiago. 2024.

[77] North J. Private Health Insurance. 2020. 10.1017/9781139026468

[78] United Nations Partnership on the Rights of Persons with Disabilities. Global situational analysis of the rights of persons with disabilities. 2022.

[79] Espinoza MA, Cabieses B, Goic C, Andrade A. The legal path for priority setting in Chile: a critical analysis to improve health planning and stewardship. Front Public Health. 2024. 10.3389/fpubh.2023.1302640. 38259787 10.3389/fpubh.2023.1302640 PMC10801194

