# Supplementary material for: Inclusive health for people with disabilities in Chile: a national health system assessment
Source: Health Res Policy Syst. 2025 Feb 20;23:22. doi: 10.1186/s12961-024-01241-4 (PMC11843747; doi:10.1186/s12961-024-01241-4)
Supplement: Supplementary file 1 — Supplementary Material 1. [file 12961_2024_1241_MOESM1_ESM.docx]

| **Additional Table 1. Civil society representation by type of organization and impairment group (n=8)** | | | | |
| --- | --- | --- | --- | --- |
| **Impairment type or disease** | **Type of organization^*^** | | | |
|  | **Of people with disabilities (n=2)** | **For people with disabilities (n=2)** ^†^ | **Of and for people with disabilities (n=1)** | **Patients’ association (n=3)** |
| Any | 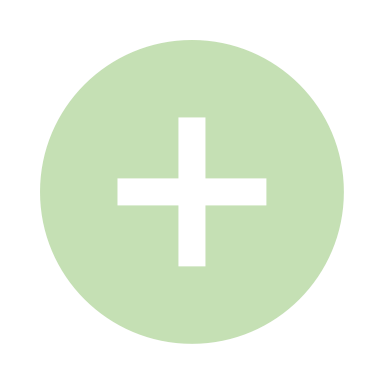 | 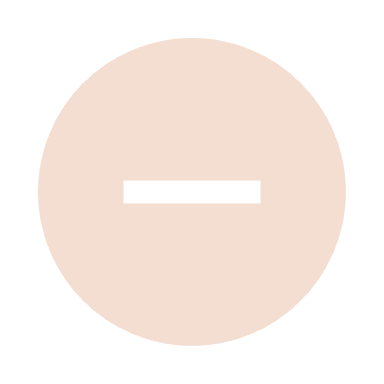 | 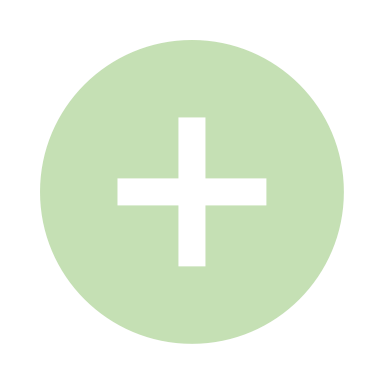 | 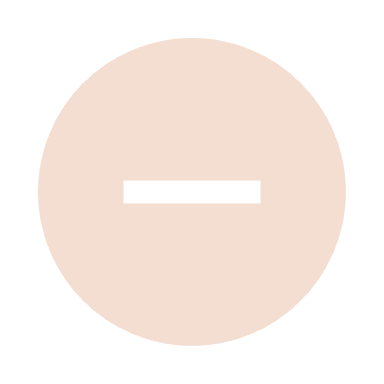 |
| Visual | 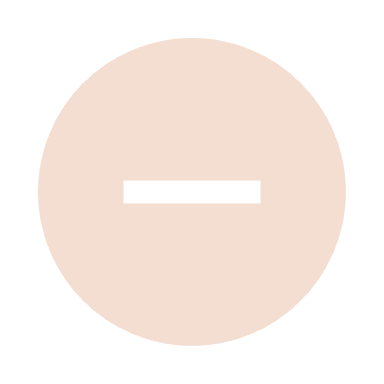 | 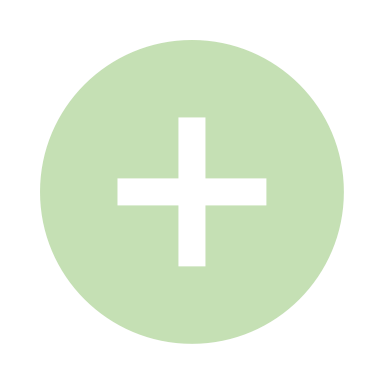 | 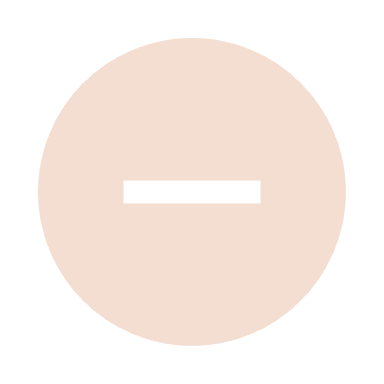 | 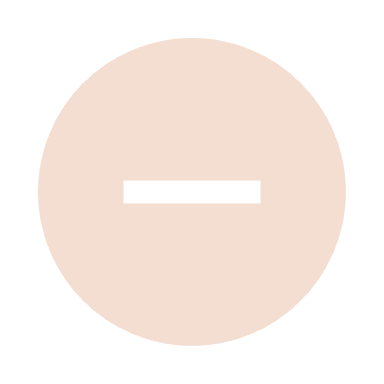 |
| Hearing | 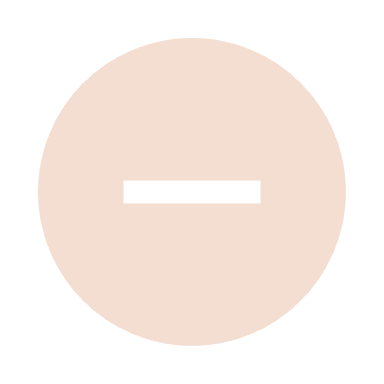 | 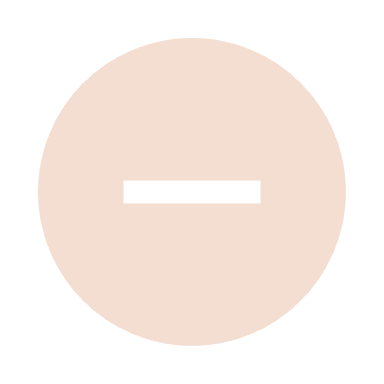 | 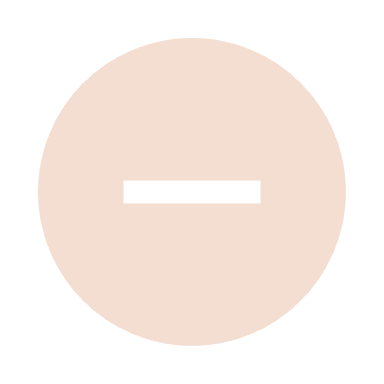 | 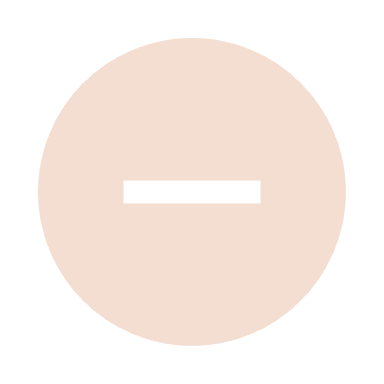 |
| Deafblindness | 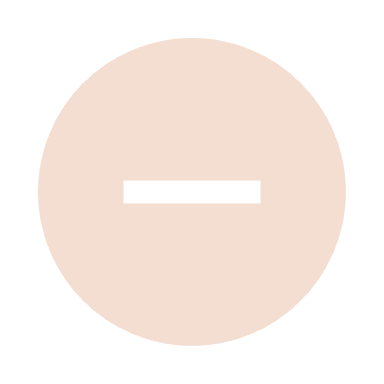 | 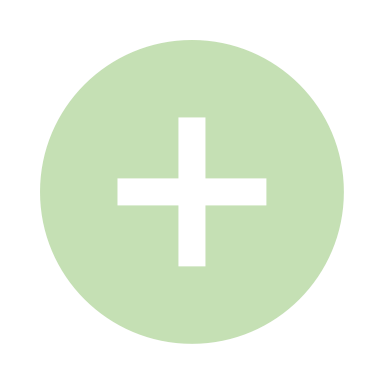 | 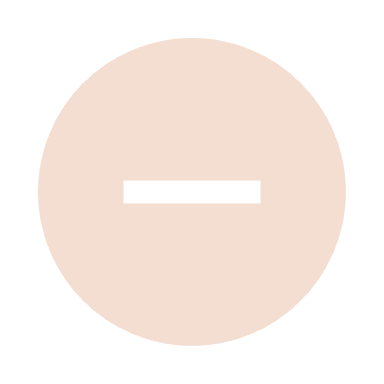 | 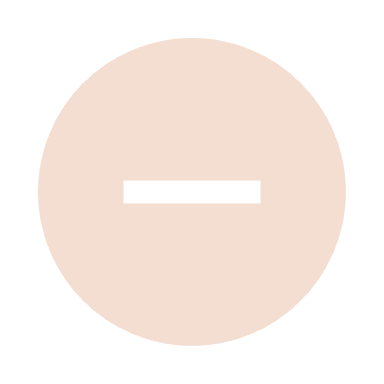 |
| Physical | 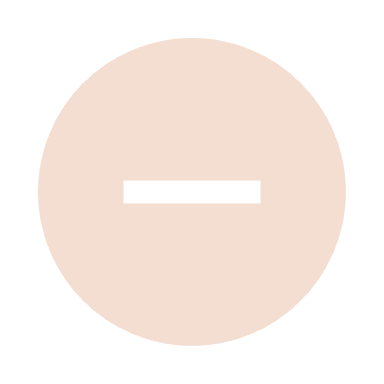 | 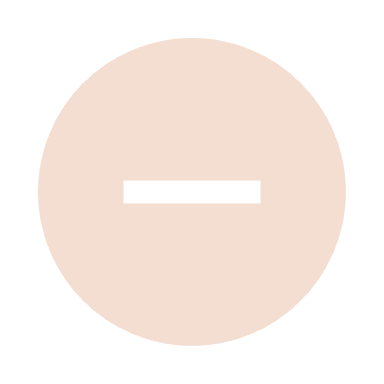 | 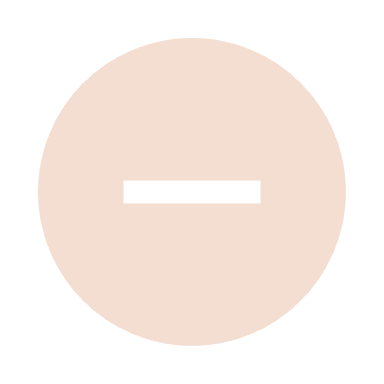 | 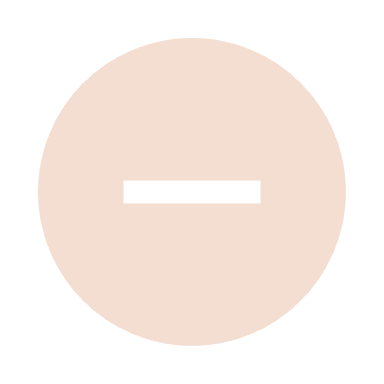 |
| Intellectual | 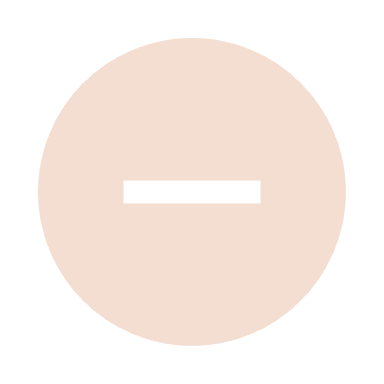 | 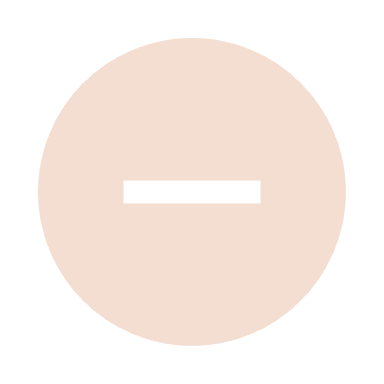 | 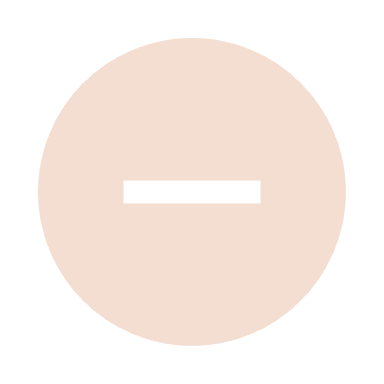 | 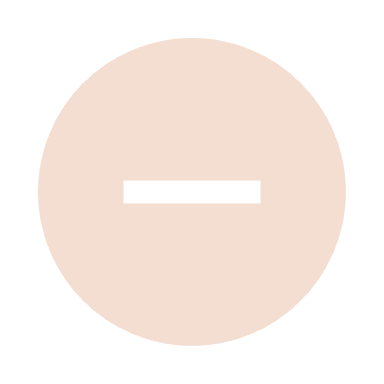 |
| Psychosocial | 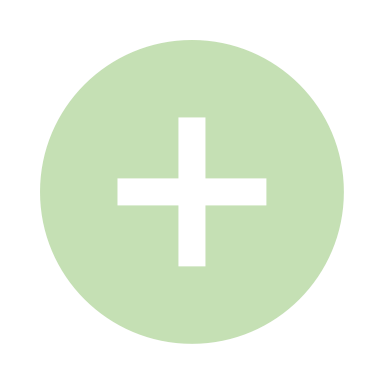 | 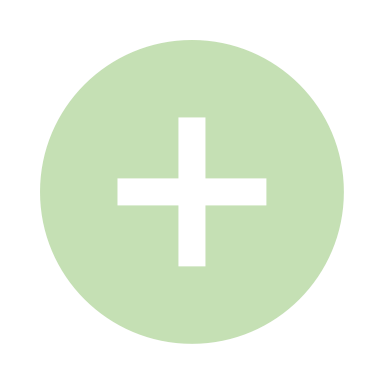 | 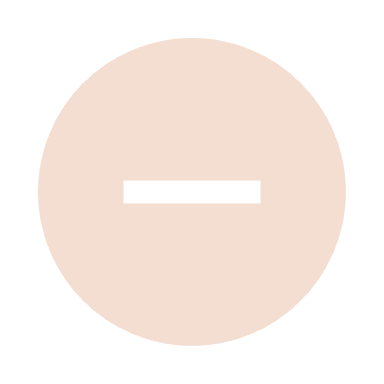 | 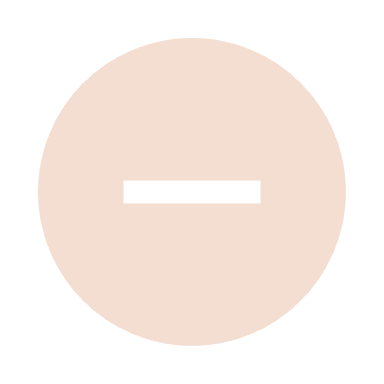 |
| Disease^‡^ | 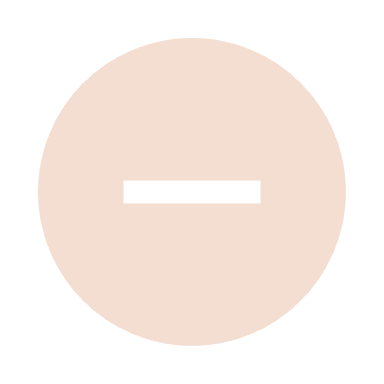 | 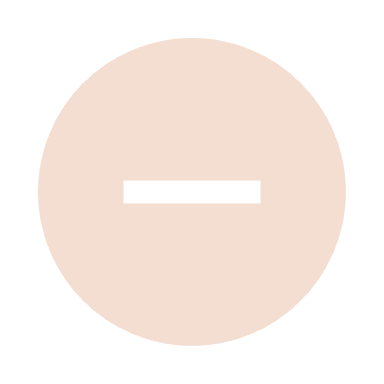 | 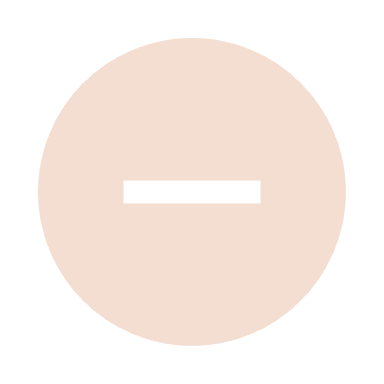 | 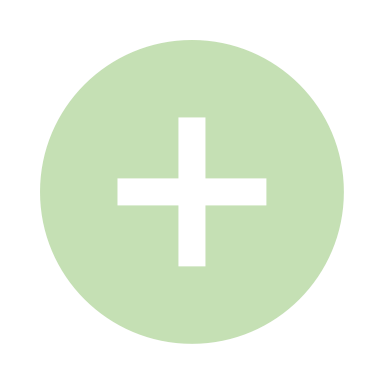 |
| ^*^According to the National Registry of and for People with Disabilities of the National Disability Agency (<http://externos.senadis.cl/catastro/>) and the National Registry of Health-related Organizations (<https://www.minsal.cl/wp-content/uploads/2021/08/Consolidado-pagina-web-28-04-2021.pdf>) . | | | | |
| ^†^One organization represented both visual disability and deafblindness. | | | | |
| ^‡^Patient associations were grouped by Fibromyalgia, Rare diseases, and Lupus. | | | | |

| **Additional Table 2. List of indicators, metric and scoring logic** | | |  |  |
| --- | --- | --- | --- | --- |
|  | **Metric and scoring logic** | | | |
| **Indicator** | **Basic/Maximum score** | **Mid-points / additional sub-criteria** | | **Lowest score** |
| 1.1 UNCRPD | Yes, ratified and adopted (1) | a) Ratified with no evidence of action (0.5) | | No (0) |
| 1.2 National Law | Yes, it protects the right to health for people with disabilities (0.33) | a) Prohibits discrimination (+0.33) and b) requires reasonable accommodations (+0.33) | | No (0) |
| 1.3 National Health Policy or Decree | Yes, national policy on health for people with disabilities exists (0.25) | a) General healthcare services (+0.25) b) Rehabilitation, AT, and specialist services (+0.25) c) Measures to implement these services (+0.25) | | No (0) |
| 1.4 National Health Sector Plan(s) | Yes, it includes people with disabilities (0.2) | a) Actions and targets for general healthcare (+0.2) and b) specialist services (+0.2) c) Basic statistics on disability and health (+0.2) d) Monitoring and evaluation of indicators (+0.2) | | No (0) |
| 1.5 National Disease Plan(s) | Yes, people with disabilities are included, and testing, treatment, and information programs are ensured (1) | | | No (0) |
| 1.6 Cross ministry governance | Yes, structure exists to coordinate work on disability inclusion (0.5) | a) MoH is included (+0.5) | | No (0) |
| 2.1 MoH leadership | A focal point/team is responsible for ensuring health access for people with disabilities (1) | | | No (0) |
| 2.2 National health sector coordination | Yes, with formal representation of persons with disabilities (individual or OPDs) in highest-level (1) | | | No (0) |
| 2.3 Pandemic preparedness structures | Yes, with formal representation of people with disabilities (individuals or OPDs) in national taskforce (1) | | | No (0) |
| 3.1 Disability inclusion budget | Yes, in MoH (or devolved levels) for department working on disability inclusion (1) | | | No (0) |
| 3.2 Reimbursement adjustments | Yes, there is a national health insurance reimbursement or there is adjusted capitation rates for services provided to some patients with disabilities (1) | | | No (0) |
| 3.3 Rehabilitation/AT budget | Yes, in MoH (or devolved levels) (1) | | | No (0) |
| 4.1 Maturity of disability and health data collection | Health information records tag people with disabilities (electronic integrated system) (1) | a) National register for people with disabilities connected to health data (0.67) b) National survey/census asks disability questions (0.33) | | Not collected (0) |
| 4.2 Quality of disability and health data collection method | All criteria fulfilled (1) | a) Valid method (0.25) and b) recent data collection (in <10 years) (0.25) c) Data is nationally representative (0.25) d) 5+ impairment types are covered (0.25) | | No criteria fulfilled (0) |
| 4.3 Maturity of disability and health data usage | Data collected is analysed, published, and used to direct policy change (1) | a) Data is analysed and published (0.5) | | Neither (0) |
| 4.4 Quality of disability and health data usage method | All criteria fulfilled (1) | a) Method is transparent and valid (0.25) b) Data is analysed and published within three years of collection (0.25) and c) the analysis is nationally representative (0.25) d) Publications and raw data are easily accessible (0.25) | | No criteria fulfilled (0) |
| 5.1 OPDs advocacy | Yes, OPDs advocate on the right to health and have been engaged in advisory roles/partnerships with the MoH (1) | | | No (0) |
| 5.2 Autonomy and awareness | In the last 10 years, people with disabilities were asked about autonomy and awareness about health in a quantitative survey or qualitative data was published in a peer-reviewed journal (1) | | | Not reported (0) |
| 5.3 Accessibility of health information | Yes, health information is available in two or more accessible formats (1) | | | <2 accessible formats (0) |
| 6.1 Health coverage | Yes, people with disabilities are fully covered for free healthcare (1) | a) Healthcare is partially covered (0.5) | | No (0) |
| - 1. Transport subsidy | Yes, available for people with disabilities including travel to medical care (1) | a) Subsidized transport but not facility dedicated services (0.5) | | No (0) |
| 6.3 Disability allowance | Yes, available to cover healthcare fees not covered by existing insurance or tax-based systems to people with moderate to severe disabilities (1) | a) Disability allowance available for some people with disabilities in the country (0.5) | | No (0) |
| 6.4 Co-payments | Yes, co-pays for services in either health insurance or taxation-based systems are waived for people with disabilities (1) | | | No (0) |
| 7.1, 7.2 & 7.3 Training of medical doctors, nurses, and CHWs | Yes, information about disability is delivered as part of their national curricula (0.33) | a) Training content covers medical and non-medical modules (+0.33) and b) is part of the core curriculum (+0.33) | | No (0) |
| 7.4 Representation of people with disabilities in health workforce | Yes, representation is in line with or greater than disability prevalence of the working age population (2% for LMICs, 4% HICs) (1) | | | No (0) |
| 7.5 Satisfaction | In the last 10 years, people with disabilities were asked about satisfaction with health services in a quantitative survey or qualitative data was published in a peer-reviewed journal (1) | | | No (0) |
| 8.1 National accessibility standards | Yes, national accessibility standards exist for healthcare facilities (1) | | | No (0) |
| 8.2 Accessibility audit | Yes, accessibility audit of health facilities has been undertaken (in <10 years) (0.33) | a) Results published in government report or peer-reviewed journal (+0.33) and b) is mandatory for all facilities to meet the accessibility standards (+0.33) | | No (0) |
| 9.1 National assessments of rehabilitation/AT | Yes, conducted in the last 10 years (1) | | | No (0) |
| 9.2 Cross-ministry coordination for rehabilitation and AT | Yes, where more than one ministry is involved (1) | | | No (0) |
| 9.3 Trained workforce for rehabilitation and AT | Above 300 physiotherapists/1,000,000 population for high-income countries (1) | | | Below the threshold (0) |
| Abbreviations: Assistive Technology (AT), Community Health Workers (CHWs), High-Income Countries (HIC), Low- and Middle-Income Countries (LMICs) Ministry of Health (MoH), United Nations Convention on the Rights of Persons with Disabilities (UNCRPD). | | | | |
